# Cognitive Mapping Based on Conjunctive Representations of Space and Movement

**DOI:** 10.3389/fnbot.2017.00061

**Published:** 2017-11-22

**Authors:** Taiping Zeng, Bailu Si

**Affiliations:** ^1^State Key Laboratory of Robotics, Shenyang Institute of Automation, Chinese Academy of Sciences, Shenyang, China; ^2^University of Chinese Academy of Sciences, Beijing, China

**Keywords:** medial entorhinal cortex, head-direction cells, conjunctive grid cells, cognitive map, attractor dynamics, path integration, monocular SLAM, bio-inspired robots

## Abstract

It is a challenge to build robust simultaneous localization and mapping (SLAM) system in dynamical large-scale environments. Inspired by recent findings in the entorhinal–hippocampal neuronal circuits, we propose a cognitive mapping model that includes continuous attractor networks of head-direction cells and conjunctive grid cells to integrate velocity information by conjunctive encodings of space and movement. Visual inputs from the local view cells in the model provide feedback cues to correct drifting errors of the attractors caused by the noisy velocity inputs. We demonstrate the mapping performance of the proposed cognitive mapping model on an open-source dataset of 66 km car journey in a 3 km × 1.6 km urban area. Experimental results show that the proposed model is robust in building a coherent semi-metric topological map of the entire urban area using a monocular camera, even though the image inputs contain various changes caused by different light conditions and terrains. The results in this study could inspire both neuroscience and robotic research to better understand the neural computational mechanisms of spatial cognition and to build robust robotic navigation systems in large-scale environments.

## 1 Introduction

One of the fundamental challenges to integrate robots into our daily life is to endow with the ability of spatial cognition. Over the past two decades, many efforts have been exerted to develop truly autonomous robot navigation systems that are able to simultaneously localize, map, and navigate without human interventions in domestic home and workplace. This line of research has been recognized as the problem of Simultaneous Localization and Mapping (SLAM) (Smith and Cheeseman, 1986; Bailey and Durrant-Whyte, 2006; Durrant-Whyte and Bailey, 2006; Thrun and Leonard, 2008) and constitutes a significant area of robotic research. The approaches to solving SLAM problem fall into two classes. The classical approach is the probabilistic one that uses the Extended Kalman Filters, the Particle Filters, etc., as the basis for robot SLAM (Thrun et al., 2005). Numerous variants of filtering algorithms have been proposed to estimate the position and the heading direction of the robot in large environments. However, most of these algorithms require costly sensors, significant computational resources, and the assumption of static environment. Although filtering algorithms provide beautiful mathematical frameworks for SLAM, they are in lack of robustness, since bad data association (i.e., the matching between perception and internal representation) can often lead them into irreversible divergence, impairing their application to dynamic and large-scale environments. For a good review, one can refer to Thrun and Leonard (2008); Dudek and Jenkin (2010).

The other approach of SLAM research is sparkled by the inspirations from neuroscience and aims to build neural network models that work in analogy to the neural circuits of spatial cognition (Zeno et al., 2016). Animals show amazing navigation ability (Klaassen et al., 2011; Tsoar et al., 2011), which has long been hypothesized to result from internal map-like representation in neural space, i.e., cognitive map of the environment (Tolman, 1948). The idea of cognitive map finds embodiment, thanks to the discovery of place cells (PCs) in the hippocampus of mammalian brains (O’Keefe and Dostrovsky, 1971; O’Keefe and Conway, 1978). A random set of place cells are recruited to encode positions in an environment and fire selectively in some restricted portions of the environment. The firing locations of place cells are randomly redistributed across environments and do not conserve metric relation. Place cells are considered to form topological maps of environments. Head direction cells (HD cells), first found in the post-subiculum (Taube et al., 1990), fire when the animal’s head points to a specific direction relative to an allocentric reference frame. Head directions cells exist in various brain regions (Taube, 2007) and provide directional information like a compass. The grid cells discovered in the dorsolateral band of the medial entorhinal cortex (dMEC) fire in multiple locations that collectively define triangular grids spanning the whole environment explored by the animal (Hafting et al., 2005). The population codes of grid cells can accurately encode animal’s spatial positions in an environment (Mathis et al., 2012). The relative spatial phases of nearby grid cells in dMEC are kept fixed across environments, up to a coherent rotation and shift (Fyhn et al., 2007). Grid cells are thought to be an important ingredient of the inner GPS in mammalian brains (Knierim, 2015). Grid cells show a rich variety of response properties. Pure positional grid cells, abundant in layer II of MEC, do not show strong modulation by movements. Conjunctive grid cells, residing mainly in layer III, V, and VI of MEC, are selective also to running directions (Sargolini et al., 2006). More recently, speed cells found in the MEC are characterized by a context-invariant positive, linear response to running speed, which is a key component of the dynamic representation of the animal’s self location in MEC (Kropff et al., 2015).

Sitting between sensory cortex and hippocampus, MEC is a converging area where grid cells, HD cells, and speed cells work together to integrate movement and sensory information. Computational models of grid cells reveal possible information processing mechanisms of MEC in forming spatial representations and provide building blocks for cognitive mapping systems (Zilli, 2012). The large majority of grid cells can be divided into three classes: oscillatory interference (OI) models, continuous attractor network models (CAN) (Fuhs and Touretzky, 2006; McNaughton et al., 2006; Burak and Fiete, 2009; Knierim and Zhang, 2012; Si et al., 2014), and self-organization (SO) models (Kropff and Treves, 2008; Si and Treves, 2013; Stella et al., 2013; Stella and Treves, 2015). OI models propose that the hexagonal grid pattern arises from the interference between velocity modulated oscillators and a theta oscillator (Burgess et al., 2007; Welday et al., 2011). The OI models are susceptible to drifting error and lack of robustness (Remme et al., 2010) and may not be robust enough for robot navigation systems. The CAN models use structured connections between cells to generate hexagonal grid firing patterns in the population level (Fuhs and Touretzky, 2006; McNaughton et al., 2006; Burak and Fiete, 2009). The grid firing pattern is a stable network state arising from the collective behavior of the neural population and lies in a continuous manifold of steady states. If coupled by velocity inputs, a continuous attractor network model of grid cells is able to propagate its network state along the manifold, perform accurate path integration, and, therefore produce single cell hexagonal grid firing patterns in the environment (Burak and Fiete, 2009). The conjunctive grid cells observed in layer III and deeper layers of MEC are modeled by a CAN that encodes position and velocity explicitly (Si et al., 2014). The conjunctive position-by-velocity representation produces intrinsically moving patterns that can be selected by velocity inputs. The movement of the pattern is not dependent on the amplitude of the velocity signal or on the firing properties of the cells and, therefore, is able to achieve robust path integration even if the connections are contaminated by noise. SO models of grid cells assume that grid cells receive broadly tuned spatial inputs, e.g., feedforward inputs from visual areas or the hippocampus. Single cell firing rate adaptation could result in triangular firing patterns (Kropff and Treves, 2008). Since the feedforward inputs function as error correcting signals, the anchoring of grid maps in the environment is strongly influenced by the inputs experienced by the animal (Si et al., 2012; Stensola et al., 2015).

Although numerous computational models of grid cells have been proposed to account for the mechanisms of integrating movement and sensory information into spatial representations, so far, only a few robot navigation systems utilize computational mechanisms of spatial cognition. In the model of RatSLAM, pose cells were developed to encode positions in large-scale environment for long-term robotic navigation tasks (Milford and Wyeth, 2008). Pose cell is a kind of abstract cell, which updates its activity by displacing a copy of the activity packet, rather than performing path integration according to the dynamics of the network. In Jauffret et al. (2015), a modulo projection of the path integration is used to generate the grid cell firing patterns. However, grid cells aid neither localization nor mapping in the mobile robot system. There is a sparse number of robot navigation systems, which use grid cell networks to do path integration and represent the pose of a robot (Sheynikhovich et al., 2009; Yuan et al., 2015; Mulas et al., 2016). Sheynikhovich et al. (2009) built a biological plausible model including visual, medial entorhinal cortex, hippocampus, and striatum to perform navigation in a simulated environment. Their model successfully reproduced many experimental results, such as rescaling of firing fields of place cells and grid cells and solved water maze task by reinforcement learning based on spatial representations. Both (Yuan et al., 2015) and (Mulas et al., 2016) used a model of pure positional grid cells in layer II of MEC (Burak and Fiete, 2009) to track the position of a robot and used visual inputs to anchor grids in the environment. They tested their system in indoor environments of dozens of meters in side. The robustness is yet to be verified in large-scale environments.

The theory of neural dynamics has been extensively adopted to model brain functions, such as memory (Treves, 1990; Mi et al., 2017), navigation (Gerstner and Abbott, 1997), and sensory integration (Zhang et al., 2016). The theory of neural dynamics has become popular in robotics to enhance the cognitive abilities of robots (Erlhagen and Bicho, 2006), such as obstacle avoidance (Xiao et al., 2017), coordinated path tracking (Xiao and Zhang, 2016), motion tracking (Ding et al., 2017), and grasping (Knips et al., 2017).

In this work, we follow the research line of neural dynamics and developed a cognitive mapping model for mobile robots, taking advantage of the coding strategies of the spatial memory circuits in mammalian brains. The key components of the proposed model include HD cells, conjunctive grid cells, and local view cells. Both HD cells and conjunctive grid cells are modeled by continuous attractor networks that operate on the same principles. More specifically, HD cells in the model represent arbitrary conjunctions of head directions and rotations of the animals. Due to the asymmetric recurrent connections and the network dynamics, intrinsically rotating patterns spontaneously emerge in the network. Angular velocity inputs to the network pick up patterns rotating with appropriate velocities and track the head direction of the robot. Similarly, conjunctive grid cells in the model encode conjunctions of positions and translations in the two-dimensional environment. The inputs from HD cells and speed cells activate a subset of the conjunctive grid cells that produce triangular patterns moving intrinsically in the neural tissue with velocity proportional to the running velocity of the robot. Both HD cells and conjunctive grid cells get inputs from local view cells, which are activated once the robot moves to a familiar scene and which provide anchoring cues. The mapping performance of the proposed model is demonstrated on a 66 km long urban car journey. Our cognitive mapping model generates a coherent map of the outdoor environment using single camera image data. The experimental results show that our model is stable and robust in large-scale outdoor environment of variable light conditions and terrains.

The major contributions in this paper are twofold. First, the neural network framework presented in this paper is based on recent experimental studies on the hippocampal–entorhinal circuits and aims to model layer III and deep layers of the MEC and the hippocampus. In the system, conjunctive HD-by-velocity cells and conjunctive grid-by-velocity cells work hand in hand to integrate movement and sensory information and build a large-scale map. Second, the neural dynamics of the hippocampal–entorhinal circuits is modeled. Provided with the inputs from local view cells, the neural dynamics of the system functions as a general mechanism for error correction and pattern completion.

This paper proceeds as follows. In Section 2, we describe the HD cell model, the conjunctive grid cell model, and other core components of the robot navigation model. The experimental setup and the resulting neural activities, rate maps, and the semi-metric topological map are presented in Section 3. Section 4 discusses the results and points to future directions, with a brief conclusion in Section 5.

## 2 Materials and Methods

In this study, we propose a cognitive mapping model that has similar regions and cell types as in the entorhinal–hippocampal circuits of mammalian brains. Localization is the key function of the proposed model and is performed by the HD cells and the conjunctive grid cells. The HD cells and the conjunctive grid cells in the model correspond to layer III, V, and VI of the MEC of mammalian brains. They integrate rotational or translational velocity and form directional or positional codes, respectively. The local view cells in the model correspond to the retrosplenial cortex or visual cortex and relay visual inputs to the MEC. Cognitive map in the system corresponds to the hippocampus.

### 2.1 Neural Network Architecture

**Figure 1:**
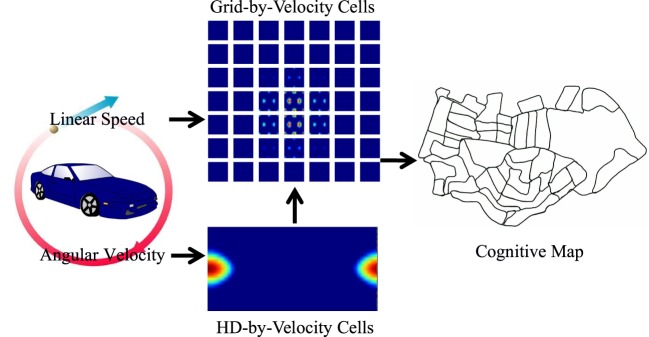
The neural network architecture of the model and the diagram of information flow. The HD-by-velocity cells, updated by angular velocity, represent head directions. The grid-by-velocity cells receiving translational velocity, converted from linear speed and the head direction representations, provide positional representation, which is in turn utilized to build a cognitive map. The acitivities of the HD-by-velocity cells and the grid-by-velocity cells are encoded by heat maps with red for high activity and blue for no activity.

The architecture of the proposed model is shown in Figure 1. The HD cells in the model form a ring attractor in the neural space. A bump of activity emerges in the network. The phase of the bump represents the robot’s real angle of the head direction in the physical environment up to a constant shift. Tuned angular velocity inputs active a subset of the HD neurons, and the bump rotates intrinsically with the same velocity as the angular velocity of the robot.

The conjunctive grid cells in the model form a torus attractor due to the periodic boundary conditions of the neural space. The phase of the grid pattern encodes the position of the robot in the physical environment up to modulo operation. The conjunctive grid cells are activated by the head direction cells and the speed cells, which are both available in the MEC of rodents. The grid pattern in the network moves with a velocity proportional to the movement of the animal in the physical environment. Thus, the HD cells and the conjunctive grid cells work hand in hand and form population codes of the head direction and the position of the robot in the environment.

The conjunctive representations of space and movement allow the networks to have stable states for all movement conditions. This coding property endows the network robustness regardless the movement conditions.

### 2.2 HD-by-Velocity Cell Model

The HD-by-velocity cells integrate angular velocity and represent one-dimensional head direction. This is achieved by conjunctive encoding of head direction and rotation.

#### 2.2.1 Neural Representation of Head Direction and Angular Velocity

Each unit in the network is labeled by its coordinate (*θ, ν*) on a two-dimensional manifold. *θ* ∈ [0, 2π) is the internal representation of head directions in the environment. Not that *θ* has periodic boundary condition. *ν* ∈ [−*L_r_, L_r_*] encodes angular velocity. Both *ν* and *θ* are dimensionless quantities in the neural space. They reflect the real angular velocity and the head direction of the robot. We design the connection weights between units to sustain a single bump of activity both in the direction dimension *θ* and in the rotation dimension *ν*. The strength of the connection from a presynaptic unit (*θ′*, *ν′*) to a postsynaptic unit (*θ, ν*) is written as

(1) $$J\left(\theta ,\nu |\theta^{\prime },\nu^{\prime }\right)=J_{0}+J_{1}\text{cos}\left(\theta -\theta^{\prime }-\nu^{\prime }\right)\text{cos}\left(\lambda \left(\nu -\nu^{\prime }\right)\right)\;,$$

where *J*_0_ < 0 is a uniform inhibition, *J*_1_ > 0 describes the interaction strength, and λ defines the spread of velocity tuning. The connection weight from the unit at *θ′* to the unit at *θ* in the direction dimension is asymmetric, since the postsynaptic unit that is maximally activated by *θ′* is not centered at *θ′* but at *θ′* + *ν′*. The asymmetry causes the bump to move along the direction dimension with a velocity determined by *ν′*.

#### 2.2.2 Network Dynamics

We consider the activity of the network driven by velocity and sensory inputs. The firing rate *m*(*θ, ν*) of the unit at coordinate (*θ, ν*) is governed by the differential equation

(2) $$\tau \dot{m}\left(\theta ,\nu \right)=-m\left(\theta ,\nu \right)+f\left(\iint D\theta D\nu J\left(\theta ,\nu |\theta^{\prime },\nu^{\prime }\right)m\left(\theta^{\prime },\nu^{\prime }\right)+I_{\nu }+I_{\mathrm{view}}\right),$$

where *I_ν_* and *I_view_* are the velocity tuned input and the calibration current injected by local view cells, respectively, which we will explain in detail below. τ is the time constant set to 10 ms. *f* (*x*) is a threshold-linear function: *f* (*x*) ≡ [*x*]_+_ = *x* when *x* > 0 and 0 otherwise. The shorthand notations are used $\int D\theta =\frac{1}{2\pi }\int_{0}^{2\pi }\;d\theta$, and $\int D\nu =\frac{1}{2L_{r}}\int_{-L_{r}}^{L_{r}}\;d\nu$.

#### 2.2.3 Angular Velocity Inputs

In order to integrate angular velocity, the activity bump in the HD network should be placed at appropriate position in the velocity dimension of the neural space. The desired bump location in the *ν* dimension corresponding to a given external angular velocity *V* is (Si et al., 2014)

(3) u(V)=arctan(τV).

Here, τ is the time constant defined in equation (2).

The velocity input to the HD-by-velocity units is simply modeled by a tuning function of Gaussian shape

(4) $$I_{\nu }\left(\nu |V\right)=I_{r}\left[1-\epsilon +\epsilon \;\text{exp}\left(-\frac{{\left(\nu -u\left(V\right)\right)}^{2}}{2\sigma_{r}^{2}}\right)\right]\;.$$

Here, *I_r_* is the amplitude of the rotational velocity input, ϵ defines the strength of the velocity tuning, and σ*_r_* is the sharpness of the velocity tuning.

#### 2.2.4 Estimation of the Head Direction

The center of the activity bump on the *θ* axis and the *ν* axis encodes the head direction and angular velocity of the robot. We define Fourier transformations to recover the head direction and the angular velocity of the robot from the network state

(5) $$\psi =∠\left(\iint m\left(\theta ,\nu \right)\;\text{exp}\left(i\theta \right)D\theta D\nu \right)\;,$$

(6) $$\phi =\frac{∠\left(\iint m\left(\theta ,\nu \right)\;\text{exp}\left(i\lambda \nu \right)D\theta D\nu \right)}{\lambda }.$$

Here, *i* is the imaginary unit, and function ∠(*Z*) takes the angle of a complex number *Z*. ψ ∈ [0, 2π) is the estimated phase of the bump in the direction axis of the neural space and corresponds to the head direction of the robot in the physical space. ϕ ∈ (−*L_r_, L_r_*) is the estimated phase of the bump in the velocity axis of the neural space and can be mapped to the angular velocity of the robot in the physical space by inverting equation (3)

(7) $$V=\frac{\text{tan}\left(\phi \right)}{\tau }.$$

Note that *L_r_* should be chosen large enough, so that the recovered velocity *V* is able to represent all possible angular velocities of the robot.

### 2.3 Grid-by-Velocity Cell Model

Now, we expand our HD-by-velocity cell model to do path integration in two-dimensional environments. To this end, grid-by-velocity cells need represent two-dimensional spatial locations and two-dimensional velocities. The units in the network are wired with appropriate connection profiles, so that hexagonal grid firing pattern is generated and translated in the spatial dimension of the neural space.

#### 2.3.1 Neural Representation of Position and Velocity

Units in the grid-by-velocity network is labeled by coordinates $\left(\vec{\theta },\vec{\nu }\right)$ in a four-dimensional neural space. $\vec{\theta }=\left(\theta_{x},\theta_{y}\right)$ represents two-dimensional positions in the environment. $\vec{\nu }=\left(\nu_{x},\nu_{y}\right)$ encodes the velocity components in the environment. We assume $\vec{\theta }$ has periodic boundary conditions, i.e., *θ_x_*, *θ_y_* ∈ [0, 2π). *ν_x_* and *ν_y_* are chosen in [−*L_t_, L_t_*].

The connection weight from unit $\left(\vec{\theta^{\prime }},\vec{\nu^{\prime }}\right)$ to $\left(\vec{\theta },\vec{\nu }\right)$ is described as

(8) $$J\left(\vec{\theta },\vec{\nu }|\vec{\theta^{\prime }},\vec{\nu^{\prime }}\right)=J_{0}+J_{k}\text{cos}\left(k\sqrt{\sum_{j\in \left\{x,y\right\}}\;||\theta_{j}-{\theta^{\prime }}_{j}-{\nu^{\prime }}_{j}|{|}^{2}}\right)\text{cos}\left(\lambda \sqrt{\sum_{j\in \left\{x,y\right\}}\;{\left(\nu_{j}-{\nu^{\prime }}_{j}\right)}^{2}}\right),$$

where the integer *k* = 2, is chosen so that the network accommodates two bumps both in *θ_x_* axis and in *θ_y_* axis. There is only one bump in each of the velocity dimensions, however. ||*d*|| is the distance on a circle: ||*d*|| = mod(*d* + π, 2π) − π, and mod(*x, y*) ∈ [0, *y*) gives x modulo y.

#### 2.3.2 Network Dynamics

Although grid-by-velocity units are organized with different manifold structure from the HD-by-velocity units, they share the same intrinsic dynamics as in equation (2)

(9) $$\tau \dot{m}\left(\vec{\theta },\vec{\nu }\right)=-m\left(\vec{\theta },\vec{\nu }\right)+f\left(\iint D\vec{\theta }D\vec{\nu }J\left(\vec{\theta },\vec{\nu }|\vec{\theta^{\prime }},\vec{\nu^{\prime }}\right)m\left(\vec{\theta^{\prime }},\vec{\nu^{\prime }}\right)+I_{\nu }+I_{\mathrm{view}}\right).$$

Note that $\int D\vec{\theta }=\frac{1}{4\pi^{2}}\int_{0}^{2\pi }\;\int_{0}^{2\pi }\;d\theta_{x}d\theta_{y}$, and $\int D\vec{\nu }=\frac{1}{4L_{t}^{2}}\int_{-L_{t}}^{L_{t}}\;\int_{-L_{t}}^{L_{t}}\;d\nu_{x}d\nu_{y}$.

#### 2.3.3 Translational Velocity Inputs

In order to perform accurate path integration, the velocity of the moving bumps in the neural space should always be kept proportional to the velocity of the robot in the physical space. The network requires tuned velocity input to pin the activity bumps at appropriate positions on the velocity axes in the neural space, so that the bumps move with the desired velocity.

The translational velocity $\vec{V}=\left(V_{x},V_{y}\right)$ of the robot is obtained by projecting the running speed to the axes of the reference frame using the HD estimated from the HD-by-velocity units (equation (5)). In the brain, running speed is encoded by the speed cells in the MEC. Given the translational velocity of the robot, the desired positions on the velocity axes in the neural space is given by $\vec{u}\left(\vec{V}\right)$ (Si et al., 2014)

(10) $$\vec{u}\left(\vec{V}\right)=\frac{1}{k}\text{arctan}\left(\frac{2\pi \tau \vec{V}}{S}\right)\;,$$

where the function arctan operates on each dimension of V →. S is a scaling factor between the external velocity of the robot in the physical environment and the velocity of the bumps in the neural space. S determines the spacing between the neighboring fields of a grid firing pattern in the environment.

The velocity-tuned inputs to the grid-by-velocity units take a Gaussian form for simplicity

(11) $$I_{\nu }\left(\vec{\nu }|\vec{V}\right)=I_{t}\left[1-\epsilon +\epsilon \;\text{exp}\left(-\frac{|\vec{\nu }-\vec{u}\left(\vec{V}\right){|}^{2}}{2\sigma_{t}^{2}}\right)\right]\;,$$

where |⋅| is the Euclidean norm of a vector. *I_t_* is the amplitude of the translational velocity input. ϵ is the strength of velocity tuning as in equation (4), and σ*_t_* the width of the translational velocity tuning.

#### 2.3.4 Estimation of the Bump Position and Localization

The center of the bumps on the neural manifold encodes spatial position and translational velocity. We use Fourier transformations to estimate the center of the bumps on the four-dimensional neural manifold.

**Figure 2:**
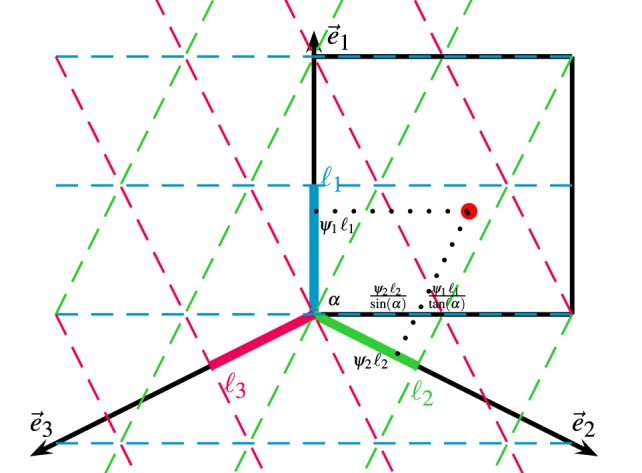
Estimation of bump position on the torus. The torus neural space of (*θ_x_*, *θ_y_*) is unfolded and shown as the black box. The grid pattern is projected by Fourier transforms along projection axes ${\vec{e}}_{1}$ and ${\vec{e}}_{2}$. The center of a bump (red dot) in the torus is represented by the phase in each projection axis. The position of the bump on the torus is recovered by mapping the phases in the projection axes to the neural space according to the trigonometric relation defined by α, which is the angle of the grid axis perpendicular to ${\vec{e}}_{2}$.

First, we project the firing activity to the projection axes defined by the grid pattern in the spatial dimensions of the neural manifold and obtain the phases of the grid pattern along the projection axes

(12) $$\psi_{j}=\frac{∠\left(\iint D\vec{\theta }D\vec{\nu }m\left(\vec{\theta },\vec{\nu }\right)\text{exp}\;\left(\mathrm{ik}\frac{{\vec{\theta }}^{T}{\vec{e}}_{j}}{\ell_{j}}\right)\right)}{k},$$

where *i* is the imaginary unit, ${\vec{e}}_{j}$ the unit direction of the *j*-th projection axis, namely ${\vec{e}}_{1}={\left[0,1\right]}^{T}$, ${\vec{e}}_{2}={\left[\text{sin}\alpha ,-\text{cos}\alpha \right]}^{T}$, ${\vec{e}}_{3}={\left[-\text{sin}\alpha ,-\text{cos}\alpha \right]}^{T}$ (see Figure 2). α, determined by the grid pattern in the torus, is the angle of the second grid axis. In our case, α = arctan(2) due to the square shape of the torus. $\ell_{j}$ is the respective wavelength of the grid pattern along the projection axis *j*, i.e., $\ell_{1}$ = 1, $\ell_{2}$ = $\ell_{3}$ = sin α.

Second, we estimate the center of the bumps in the spatial dimensions of the neural manifold according to the trigonometric relation between the projection axes and the spatial axes. As shown in Figure 2, the center of the bumps in the *θ_y_* dimension is simply

(13) $${\hat{\theta }}_{y}=\psi_{1}\ell_{1}.$$

By mapping the scaled phase $\psi_{1}\ell_{1}$ and $\psi_{2}\ell_{2}$ to the *θ_x_* direction, the center of the bumps in the *θ_x_* dimension is given by

(14) $${\hat{\theta }}_{x}=\frac{\psi_{2}\ell_{2}}{\text{sin}\alpha }+\frac{\psi_{1}\ell_{1}}{\text{tan}\alpha }.$$

The phases estimated in equation (12) are constrained by the periodic boundary conditions. In order to estimate the true position of the robot in the environment, the phases should be unwrapped by considering the phases of two consecutive time steps. After unwrapping, the cumulative phases are used in equations (13) and (14) to estimate the position of the robot

(15) $$\hat{x}\left(t\right)=\frac{S}{2\pi ∕k}{\hat{\theta }}_{x}\left(t\right),$$

(16) $$\hat{y}\left(t\right)=\frac{S}{2\pi ∕k}{\hat{\theta }}_{y}\left(t\right),$$

where ${\hat{\theta }}_{x}\left(t\right)$ and ${\hat{\theta }}_{y}\left(t\right)$ are the cumulative phases after unwrapping at time t. The scaling factor $\frac{S}{2\pi ∕k}$ is the ratio between the velocity of the robot in the physical environment and the velocity of bumps in the network.

To estimate the center of the bumps in the velocity dimensions, the same method as equation (6) is used in the following form

(17) $$\phi_{j}=\frac{∠\left(\iint D\vec{\theta }D\vec{\nu }m\left(\vec{\theta },\vec{\nu }\right)\;\text{exp}\left(i\lambda \nu_{j}\right)\right)}{\lambda },$$

where *j* ∈ {*x, y*}. ϕ*_j_* ∈ (−*L_t_, L_t_*) is the position of the bump on the axes *ν_j_*.

According to the mapping between the velocity of the bumps in the neural space and the velocity of the robot, the velocity of the robot in the environment is given by

(18) $$V_{j}=\frac{S\text{tan}\left(k\phi_{j}\right)}{2\pi \tau }.$$

Note that *L_t_* should be chosen large enough to cover the maximal translation velocity experienced by the robot.

### 2.4 Estimating Angular and Translational Velocity from Visual Inputs

The angular velocity and the translational speed of the robot are estimated by matching two consecutive frames from the camera, according to the method described in Milford and Wyeth (2008). The estimated angular velocity is utilized by the HD-by-velocity units to form HD representations (equation (4)). The translational velocity is obtained by combining the HD estimation from the HD-by-velocity network and the translational speed estimated from the image sequence, and is fed to the grid-by-velocity network to perform path integration (equation (11)).

### 2.5 Calibration from Local View Cells

Since movement estimation is subject to noise, both the head direction cell network and the grid cell network need calibration by visual inputs. Following Milford and Wyeth (2008), we extract local view templates from images to encode the scenes observed from the camera. If a view is sufficiently different from previously observed views, a new view cell is added to the system, together with the view template and the estimated bump positions ψ, ${\hat{\theta }}_{x}$ and ${\hat{\theta }}_{y}$. If the robot comes back to a previously visited location, and a familiar view reappears, the corresponding local view cell is activated and provides inputs to the networks. We model the current injected from a view cell to the HD cell network as

(19) $$I_{\mathrm{view}}\left(\theta \right)=I_{d}\text{exp}\left(-\frac{||\theta -\psi |{|}^{2}}{2\sigma_{d}^{2}}\right)\;,$$

where *I_d_* is the amplitude of the injected current to the HD cell network, ψ is the associated phase with the view template, σ*_d_* is sharpness of the Gaussian tuning.

For the conjunctive grid cell network, the input from a view cell is given by

(20) $$I_{\mathrm{view}}\left(\vec{\theta }\right)=I_{p}\left(\frac{1}{3}\sum_{j=1}^{3}\;\text{cos}\left(k\frac{{\left(\vec{\theta }-\hat{\vec{\theta }}\right)}^{T}{\vec{e}}_{j}}{\ell_{j}}\right)+C\right)\;,$$

where *I_p_* is the amplitude of the injected current, *C* is a constant to adjust the baseline, $\hat{\vec{\theta }}$ is the phase associated with the local view template (see equations (13) and (14)), and other parameters are the same as in equation (12). Note that the injected current from a local view cell depends only on *θ*→, and is the same for different *ν*→.

### 2.6 Neural Network Parameters

The HD-by-velocity units are regularly arranged on a rectangular grid in the two-dimensional manifold of direction and rotation, with 51 units in the direction dimension and 25 units in the rotation dimension. All together, there are 1,275 HD-by-velocity units. *L_r_* = 0.0095 is sufficient to represent the maximal angular velocity of the vehicle.

The grid-by-velocity units are regularly arranged on a rectangular grid in the four-dimensional neuronal manifold of position and velocity. There are 15 units in each of the positional dimensions and 7 units in each of the velocity dimensions, resulting in 11,025 grid-by-velocity units. To represent the maximal experienced translational velocity of the robot, *L_t_* is chosen to be 0.3.

Table 1 summarizes the parameters used in the model.

**Table 1:** Values of the parameters used in the system.

Parameter	*J*_0_	*J*_1_	*λ*	*L_r_*	*I_r_*	*ϵ*	*σ_r_*	*J_k_*	*k*	*L_t_*	*S*	*I_t_*	*σ_t_*	*I_d_*	*σ_d_*	*I_p_*	C
Value	−60	50	0.8	0.0095	50	0.8	0.012	50	2	0.3	30	60	0.1	60	2.19	200	0.5

### 2.7 Map Representation

A map of the environment can be formed by reading out the spatial codes in the grid-by-velocity network. We could resort to place cell models (Solstad et al., 2006; Si and Treves, 2009; Cheng and Frank, 2011) to build cognitive maps. In this study, we simply adopt the experience map representation (Milford and Wyeth, 2008), which is a topological map storing the spatial positions and their transitions measured from visual odometry. A graph relaxation algorithm (Duckett et al., 2002) is applied to optimize the topological map when the robot comes back to a familiar location and closes a loop (Milford and Wyeth, 2008).

### 2.8 Implementation of the Cognitive Mapping Model

**Figure 3:**
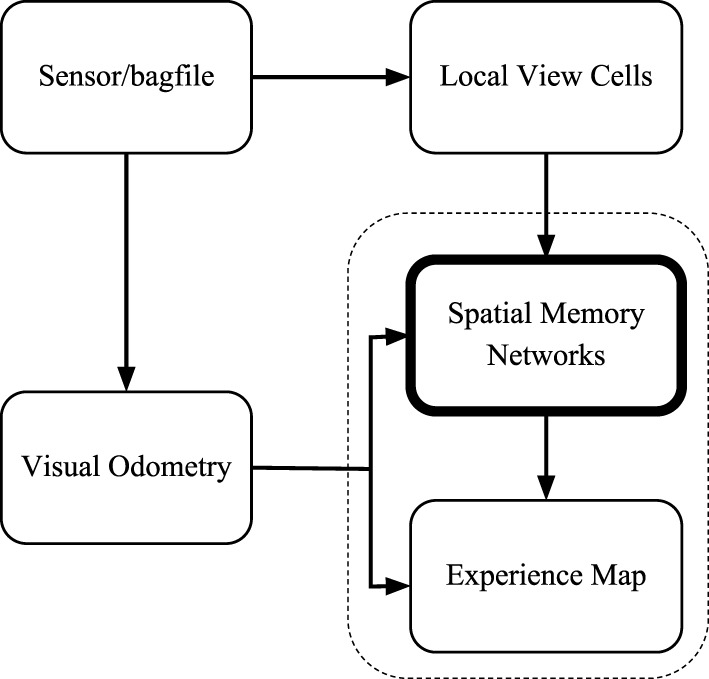
The software architecture of the cognitive mapping model. The sensor/bagfile node provides camera images. Velocities are estimated by the visual odometry node. The local view cell node analyzes whether the current view is new or not. Path integration and decisions of creating links and nodes are performed by the spatial memory network node. The topological map is generated by the experience map node.

The proposed cognitive mapping model is implemented in the C++ language and is run in the Robot Operating System (ROS) Indigo on Ubuntu 14.04 LTS (Trusty). We organize the software architecture of our cognitive mapping model into five nodes (Figure 3), similarly as that of the open RatSLAM system (Ball et al., 2013), for the ease of reusing their visual processing algorithm and comparing with their results.

The visual odometry node measures the angular velocity and the translational speed according to the changes of visual scenes. It receives frames from the ROS either from a camera or from stored data in a bagfile.

The local view cell node determines whether the current scene is new or is a familiar one. It provides calibration current to the networks in the spatial memory network node.

The spatial memory network node includes the head-direction cell network and the conjunctive grid cell network. This node receives two types of ROS messages as inputs: odometry and view templates. As shown in Section 2.2.3, 2.3.3, and 2.5, the HD cell network and the conjunctive grid cell network integrate velocity information and visual information to form neural codes of the pose of the robot. The spatial memory network node also makes decisions about the creation of nodes and links in the experience map and sends ROS messages of graph operations to the experience map node.

The experience map node reads out the neural codes of the conjunctive grid units and represents the key locations of the environment as the vertices in a topological graph. A vertex stores both the position estimated from the conjunctive grid units and the odometry relative to its previous vertex in the graph. On loop closure, a topological map relaxation method is used to find the minimum disagreement between the odometric transition information and the absolute position in the topological map space. When the position encoded in the conjunctive grid cell network is far enough from the position of the previous vertex, the spatial memory network node would inform the experience map to create a new vertex and a new edge linking to the previous vertex.

**Figure 4:**
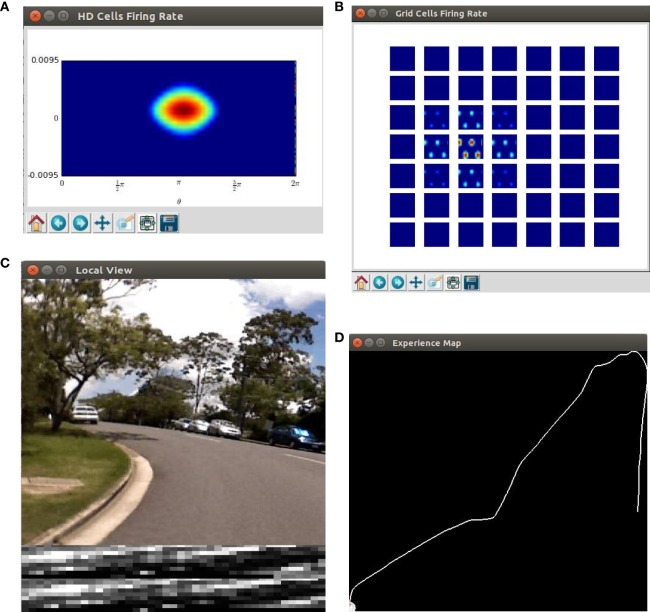
Screenshots of the cognitive mapping model. **(A)** The neural activity of the HD-by-velocity units is depicted as a heat map, with warm colors for high activities and cool colors for acitivities diminishing to zero; **(B)** the activity of the conjunctive grid-by-velocity units is shown as heat maps, and each panel corresponds to a subspace of the neural manifold with the same velocity label. The same color code as in **(A)** is used; **(C)** the video frame (top) is the visual input to the robot. A visual feature map is constructed to represent the current scene (bottom). The best matched template is shown in the middle; **(D)** experience map showing paths with nodes and links.

We write python scripts to visualize the live state of our robot navigation system. The key components of the system are shown, including the real time neural activity of the HD cells and the conjunctive grid cells (Figures 4A,B), the image of the scene and the local view templates (Figure 4C), as well as the current experience map (Figure 4D).

### 2.9 St Lucia Suburb Dataset

The St Lucia Suburb dataset was first used in Milford and Wyeth (2008). The dataset was gathered in the suburb area of St Lucia in Brisbane, Australia by a vehicle. The vehicle was driven around the road network, visiting every street at least once. The trajectory is 66 km long, finished within about 100 min at typical driving speed up to 60 km/h. The trajectory spans an area of 3 km by 1.6 km in East–West and North–South direction, respectively. The street views were recorded in 10 frames/s into a video of resolution 640 × 480 pixels by a laptop’s built-in web-cam mounted on the roof of the vehicle. The GPS information of the vehicle was not collected, however.

**Figure 5:**
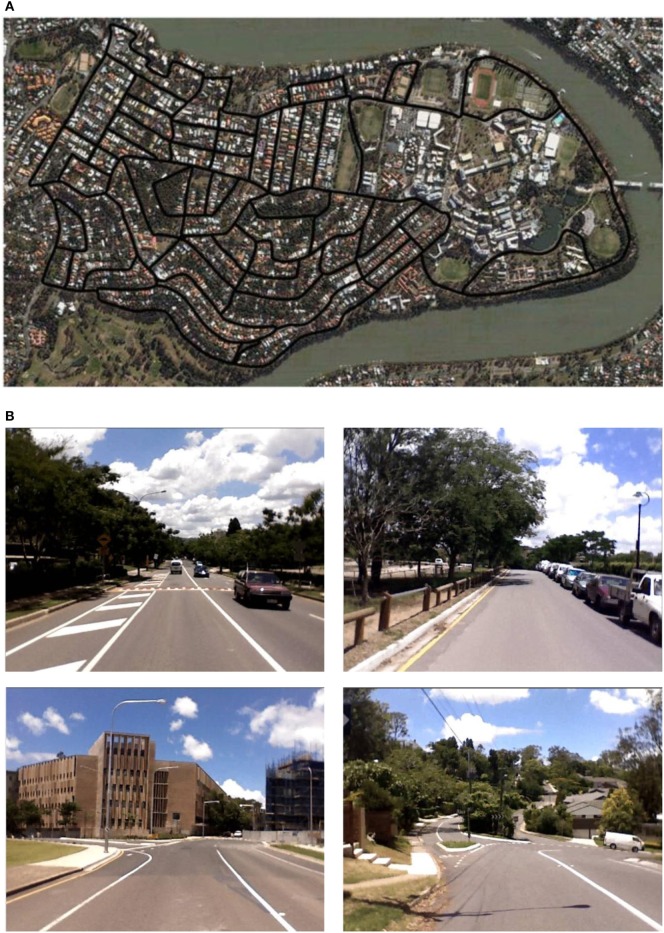
The complex environment of St Lucia. **(A)** The map of St Lucia from a bird’s eye view. Black lines are the major road network in St Lucia urban area. The area is approximately 3.0 km by 1.6 km in size; **(B)** example scenes experienced by the robot in St Lucia. Panel **(A)** reused from http://www.itee.uq.edu.au/think//filething/get/246/MichaelFig3.png.

St Lucia is a very challenging environment to map due to its big variability and fairly large scale. There are 51 inner loops of varying size and shape, and more than 80 intersections in the road network (Figure 5A). The roads vary from busy multilane streets to small tracks, the terrains range from flat plains to steep hills, and the light conditions change a lot as well (Figure 5B).

## 3 Results

We tested our cognitive mapping model on the St Lucia dataset (Ball et al., 2013). Our cognitive mapping model is run on a personal computer with 3.4 GHz six-core Intel i7 processor and 64 GB memory. The program is paralleled using OpenMP. Video S1 in Supplementary Material shows the mapping process.

### 3.1 Neural Representation

**Figure 6:**
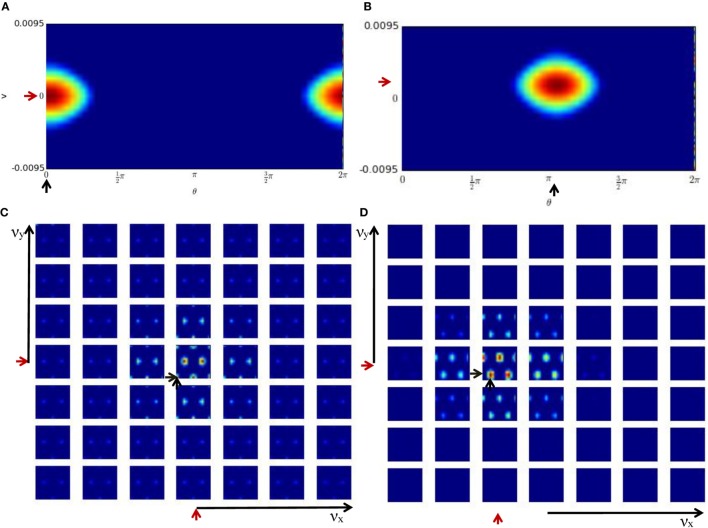
Population activities of the units in the cognitive mapping model. The activity of each unit is color-coded from red to blue for the maximal activity to zero activity. **(A)** The population activity of the HD-by-velocity units in the beginning of the experiment. The horizontal axis is the direction axis *θ*, and the vertical axis is the velocity axis *ν*. Positive (negative) *ν* represent counterclockwise (clockwise) rotation. A localized bump pattern emerges in the network. At his moment, the center of the bump is at (0, 0), indicated by the black and red arrows in the direction and velocity dimension, respectively; **(B)** the activity of the HD-by-velocity network in the middle of the experiment. The center of the bump is at (3.25, 0.0069); **(C)** the population activity of the grid-by-velocity units in the beginning of the experiment. The four-dimensional neural activity is sliced in the *ν_x_* and *ν_y_* dimensions into 49 planes shown by each panel, with increasing *ν_x_* from left to right and increasing *ν_y_* from bottom to top. Each panel depicts the activity of the units with the same velocity label, with horizontal axis for *θ_x_* and vertical axis for *θ_y_*. The center of the bumps is marked by the short black arrows in the spatial dimensions and short red arrows in the velocity dimensions; **(D)** the activity of the grid-by-velocity network in the middle of the experiment. The center of the bumps is at (3.85, 3.91, −0.0889, −0.0045) in the 4D neural space (*θ_x_*, *θ_y_*, *ν_x_*, *ν_y_*).

Figures 6A,C show the activity of the HD-by-velocity units and the conjunctive grid-by-velocity units in the beginning of the experiment, when the robot is stationary. In the HD-by-velocity network, a single bump of activity emerges. Since the angular velocity is zero at this time, the bump is centered in the velocity dimension (red arrow in Figure 6A). The position of the bump in the direction dimension (black arrow in Figure 6A) encodes the direction of the robot in the environment. At this moment, the activity of the grid-by-velocity units is also centered in the center of the velocity dimensions (red arrows in Figure 6C) due to the zero translational velocity input. In spatial dimensions, the same grid pattern sustains, with peak activity decays gradually when the velocity labels of the units are away from the center. The phase of the grid pattern (short black arrows in Figure 6C) encodes the position of the robot in the environment.

One example of the network states in the middle of the experiment is displayed in Figures 6B,D. The activity bump of the HD-by-velocity network is centered at (3.25, 0.0069) (Figure 6B), which means that the current HD angle of the robot is encoded as 186.21°, and meanwhile, the robot is rotating counterclockwise at 0.69 rad/s in the physical environment. At this moment, the activity pattern of the grid-by-velocity network is centered at *ν_x_* = − 0.0889 and *ν_y_* = − 0.0045 in the velocity dimensions, corresponding to the movement of the robot in *V_x_* = − 11.43 and *V_y_* = − 0.57 m/s.

### 3.2 Pattern Completion with Visual Feedback

**Figure 7:**
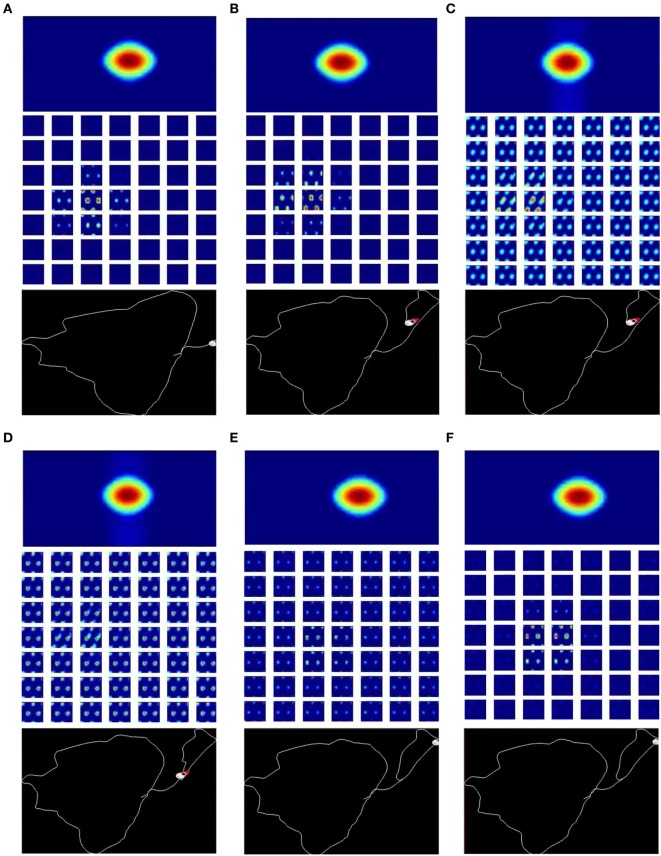
Calibration of the network states during loop closure by the help of visual feedback from the local view cells. There are three parts in each panel: the activity of the HD-by-velocity network (top), the activity of the grid-by-velocity network (middle), and the corresponding experience map at the time (bottom). **(A)** The robot drives along a path for the first time. The activity bump of the HD-by-velocity network is centered at (3.3763, 0.0023) and the bumps of the grid-by-velocity network are at (1.1781, 5.7298, −0.1027, −0.0191); **(B)** before the robot revisits a familiar place, the center of the bump of the HD-by-velocity network moves to (3.0973, 0.0021) and the center of the bumps of the grid-by-velocity network (bottom) is at (2.2176, 2.3562, −0.1272, −0.0158). **(C)** The robot revisits the same place as in **(A)** and starts to close a loop. The local view cells inject currents to the HD-by-velocity network and the grid-by-velocity network, and create weaker bumps at the same phases as the network states in **(A)**; **(D)** after multiple continuous current injections, the networks retrieve similar phases as those when the robot first visited the place; **(E)** once the robot enters a new area, the local view cells become inactive, and the network states stat to sharpen. **(F)** After loop closures, the network states are modulated by the velocity inputs. The bumps revert to their localized shapes.

The network states are calibrated by the cues provided by the local view cells. The calibration is achieved by the pattern completion mechanism of the attractor networks. Figure 7 and Video S2 in Supplementary Materials show an example of pattern completion process during loop closure. In Figures 7A,B, the robot traverses a path for the first time, while the networks integrate velocity inputs, forming localized bumps that encode the head direction and the position of the robot. When the robot closes a loop and revisits familiar places (Figures 7C,D), local view cells get activated and provide strong cues to the networks. Weak bumps emerge at the phases associated to the local view cells and quickly become the dominating bumps in the network, retrieving phases similar to the phases when the robots first visited this place (Figures 7A vs. 7D). Once the robot moves to unfamiliar areas, local view cells become inactive, and the bumps revert to their localized shapes (Figures 7E,F).

### 3.3 Cognitive Map

**Figure 8:**
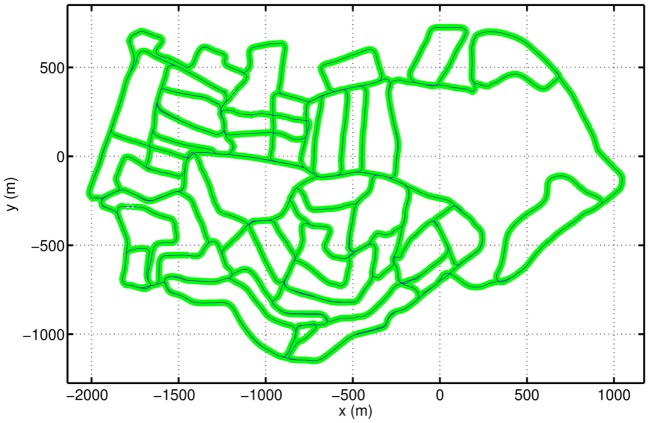
The semi-metric cognitive map of St Lucia formed in the cognitive mapping model. The green thick line comprises topological graph vertices, and the blue thin line consists of links between connected vertices.

The cognitive map created by the mapping system is shown in Figure 8. The thick green line is composed of the vertices of the topological graph. The positions of the vertices are determined by the conjunctive grid cells and graph relaxation. The link between two related vertices is shown by the fine blue line. Since the physical distance between two topological vertices is considered, the experience map is actually a semi-metric topological map, which can be compared visually with the ground truth map (Figure 5A) by naked eyes. The cognitive map conserves the overall layout of the road network of the environment. The cognitive map correctly represents all loop closures and intersections, although the orientation and length of the path are slightly different from the ground truth map. Overall, the map built by our method is consistent with the true map of the environment, and of similar quality as compared with that obtained in Ball et al. (2013).

### 3.4 Firing Rate Maps

**Figure 9:**
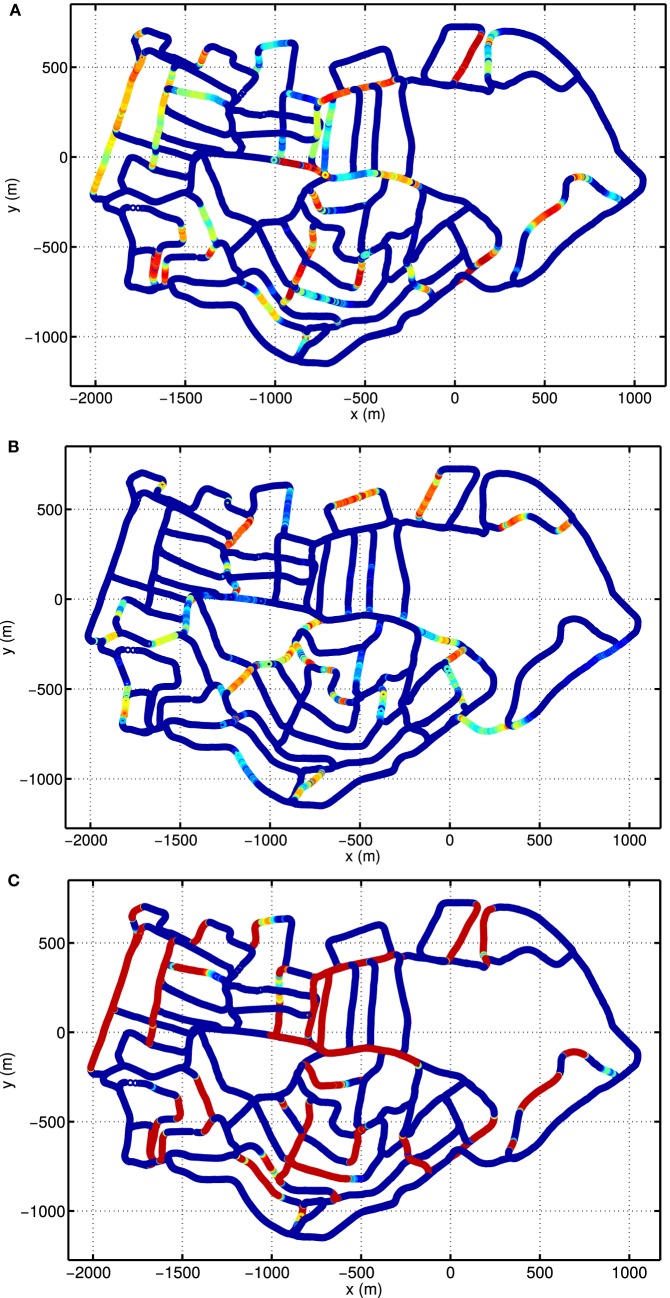
Firing rate maps of example HD-by-velocity units. In each panel, the firing rate is plotted at the locations in the experience map. Firing rate is color-coded by the same jet colormap, i.e., red for high firing rate and blue for zero firing rate. **(A)** The firing rate map of the unit at (0, 0) in the (*θ, ν*) manifold; **(B)** The firing rate map of the unit at (π, 0.0024), which has the opposite preference in head direction as the unit in **(A)**; **(C)** The total activity of all the units with the same label *θ* = 0, i.e., summing firing rates of all the units along the dimension *ν* at *θ* = 0.

To see the responses of single units, we show the activities of example units on top of the cognitive map. Figure 9A depicts the firing rate map of the HD-by-velocity unit with label (0, 0) in the neural manifold. It fires strongly when the robot moves in broad directions close to southwest. The unit responses to a wide range of directions, since the width of the bump in the HD-by-velocity network is quite big, covering about 70° in head directions. The unit does not fire on many of the roads parallel to the southwest direction, since on those roads the robot moves northeast, i.e., opposite to the preferred firing direction of the unit. This can be confirmed by the firing rate map of a unit with opposite preferred head direction, shown in Figure 9B. This unit has the label (π, 0.0024). This unit fires in broad directions close to northeast, and its firing rate map does not overlap with that of the unit in Figure 9A, meaning that it keeps silent when the unit in Figure 9A fires. This unit prefers a counterclockwise rotational velocity of 0.24 rad/s. Therefore, on bending paths, when the robot turns counterclockwise at about northeast directions, its firing rate always increases first and then decreases, which means that the head direction and the angular velocity of the robot become close to and away from the preferred conjunction of direction and rotational velocity of this unit. Figure 9C shows the firing rate map of the HD units with label 0 in the dimension *θ*. The firing pattern in Figure 9C indicates these units encode quite large range of directions centered at southwest. In local regions, these cells conserve consistent preference for directions.

**Figure 10:**
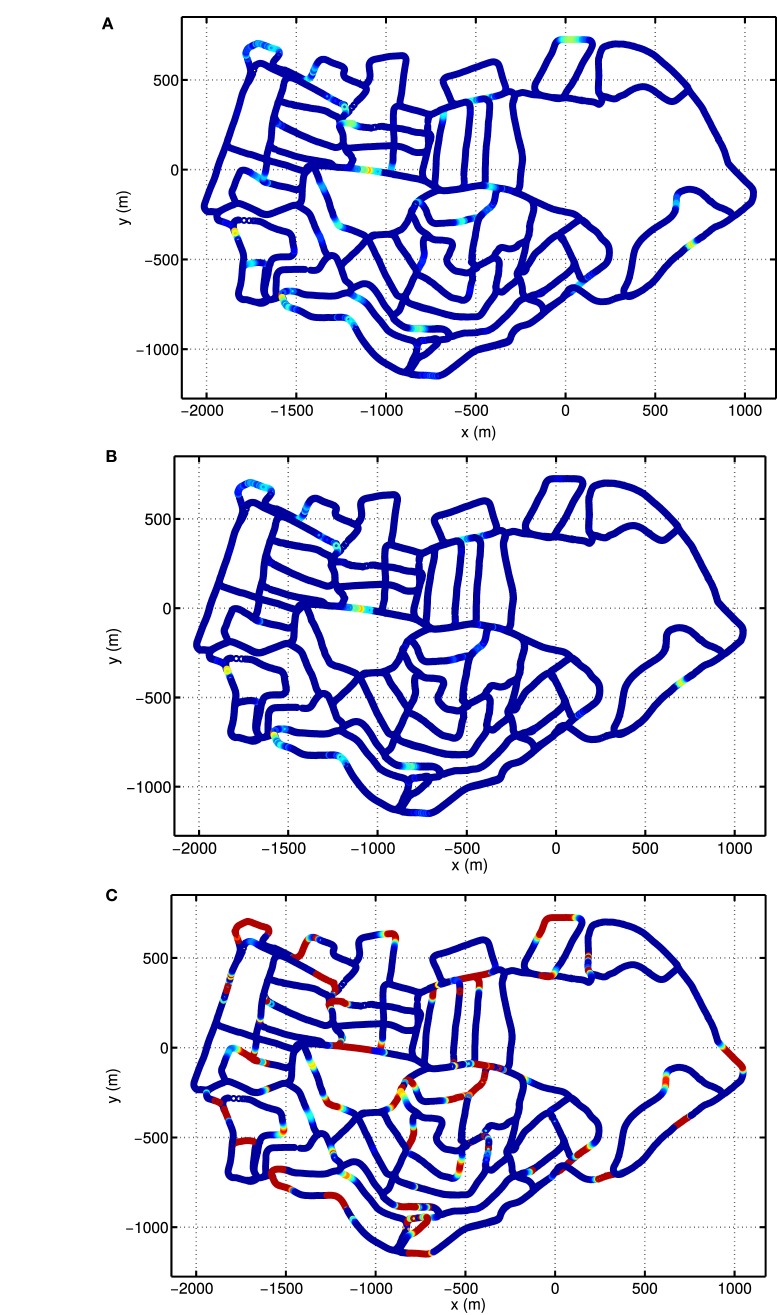
Firing rate maps of example conjunctive grid-by-velocity units. In each panel, firing rate is plotted at the locations in the experience map. For all panels, the same colormap is used to code firing rate, red for high firing rate and gradually changing to blue for firing rate decreasing to zero. **(A)** The firing rate map of the unit at (π, π, 0, 0) in the (*θ_x_*, *θ_y_*, *ν_x_*, *ν_y_*) manifold; **(B)** the firing rate map of the unit at (π, π, 0.1, 0.1); **(C)** the map of the total activity of the units at (π, π) in the (*θ_x_*, *θ_y_*) manifold, which sums the firing rates of the units along the dimensions *ν_x_* and *ν_y_*.

Due to the periodic boundary conditions of the grid pattern in the conjunctive grid-by-velocity cell network, each grid unit fires at multiple distinct locations in the environment. Figures 10A,B give the firing rate maps of two example grid units in the network with the same spatial label but different velocity labels. In each of these firing fields, the firing rate always gradually increases when the robot moves closer to the center of the firing field and decreases bit by bit when the robot leaves the field center. The firing fields of the two example grid units overlap a lot, since they have the same spatial preference. The difference in firing field comes from the fact that the grid unit in Figure 10B prefers high translational velocity, however, the grid unit in Figure 10A prefers low translational velocity. Therefore, the grid units in Figure 10A fires in many locations close to the turns of the road, where the robot has to slow down significantly. The total activity of the grid units, which share the same spatial preference as the units in Figures 10A,B is shown in Figure 10C. The firing map is composed of many distributed firing fields, but they are lack of global grid structure. This is similar to the fragmented firing pattern observed in hairpin maze (Derdikman et al., 2009), where the animal also runs on linear paths. Note that the distance between the grid fields of the units in the network is much larger than the typical spacing observed in animal experiments. This is due to the fact that the velocity input to the model is defined in the visual space of the input images and is different from the actual velocity of the robot.

## 4 Discussion

We proposed a robust cognitive mapping model, which integrates movement and sensory information and forms stable spatial representations of the environment. We demonstrated that our cognitive mapping model using only visual sensory information could successfully build a coherent topological map of large-scale outdoor environment from an open-source dataset of 66 km urban car journey (see Video S1 in Supplementary Materials).

There are two major contributions in this study. First, the network architecture of our cognitive mapping model is consistent with the synaptic organization of the MEC. Our model is built upon layer III and deep layers of the MEC. Layer III and deep layers of the MEC may be the main site to perform key computations for navigation, and the layer II of the MEC may merely relays information to the hippocampus and functions as the output of the MEC. Supporting experimental evidence shows that there exist strong projections from deep layers to superficial layers of the MEC. HD cells, conjunctive grid-by-head-direction cells, and speed cells are abundant in layer III and deep layers of the MEC, and encode direction and speed information needed in path integration. However, layer II of the MEC contains mainly pure positional grid cells. Second, our cognitive mapping model provides coherent robust mechanisms to perform path integration. Both the HD cells and the conjunctive grid cells in the model integrate motion and perception information using the same principle. The performance of the model is manifested by experiments in large-scale natural environment. Compared with the pose cell network in Ball et al. (2013), our model achieves similar mapping results as those shown in Ball et al. (2013). Although our model reuses some of the vision and map operation methods of Ball et al. (2013), the core components of the HD cell network and the conjunctive grid cell network in our system are fundamentally different from the pose cell network. In our model, path integration is performed by the intrinsic attractor dynamics driven by velocity inputs, rather than simply displacing the copy of the activity package. In the proposed model, the convergence process of local view calibration is also performed by the dynamics of the attractors, instead of global inhibition.

The grid cells in our model does not express grid-like firing pattern in large-scale environment (Figure 10). Meanwhile, the HD cells in the model fire at a broad range of directions (Figure 9). This is due to the fact that local view cells anchor grids to local cues and alters the firing patterns of the units. It has been shown that in environments with abundant local cues, such like corridors, grids cells show fragmented firing maps (Derdikman et al., 2009). It is much harder for animals to form a globally consistent metric map in large than in small environments. Indeed, as shown in Carpenter et al. (2015), grid cells in rats acquire globally coherent representations after repeated exploration, while initially grid maps are only locally consistent.

Several potential limitations exist in the current study. First, the number of units in the system is large and requires substantial amount of computational resources. Second, place cell network is not included in our system. The function of place cells is not to provide a metric map. Experiments showed that in large-scale environments, place cell has multiple irregularly spaced place fields (Rich et al., 2014; Liu et al., 2015). A metric map is necessary if direct interpretation is preferred. For robotic applications, it would suffice to have distributed neural representations if the robots are able to distinguish different locations of the environment.

For future research, we plan to include a place cell network, which integrates multiple grid cell modules with diversity of spacings and orientations, and investigate its function in path planning and reward-based learning. We will also investigate neurobiologically inspired algorithms to improve the computational efficiency of the system for robot navigation.

## 5 Conclusion

In summary, conjunctive space-by-movement attractor network models are proposed in this paper to achieve cognitive mapping in a large-scale natural environment. Head direction cells and conjunctive grid cells work on the same principle, and represent positions, head directions, and velocity at the same time. Our model facilitates to reach a better understanding of the neural mechanisms of spatial cognition and to develop more accurate models of the brain. Furthermore, it inspires innovative high performance cognitive mapping systems, which are able to function in dynamical natural environment with long-term autonomy.

## Author Contributions

TZ and BS conceived the model, performed experiments, analyzed the results, and wrote the paper.

## Conflict of Interest Statement

The authors declare that the research was conducted in the absence of any commercial or financial relationships that could be construed as a potential conflict of interest.

## Supplementary Material

Video S1 Mapping process.

Video S2 An example of pattern completion process.
