# Supplementary material for: Cognitive Mapping Based on Conjunctive Representations of Space and Movement
Source: Front Neurorobot. 2017 Nov 22;11:61. doi: 10.3389/fnbot.2017.00061 (PMC5703018; doi:10.3389/fnbot.2017.00061)
Supplement: Supplementary file 3 [file Presentation_1.PDF]

Cognitive Mapping Based on Conjunctive Representations of Space and Movement

Taiping Zeng [1,2] and Bailu Si [1,*]
*Correspondence:
Bailu Si
sibailu@sia.ac.cn

1 SUPPLEMENTARY VIDEOS

1.1 Supplementary Video 1

Localization and map building in the St Lucia data set. The population activities of the HD-by-velocity cells (top left) and the grid-by-velocity cells (top right) are shown as heat maps on their neural manifolds. The visual input to the system, the local view template and the matched template are shown in lower right. And in lower left is the constructed cognitive map. The whole experiment is shorten into 50 seconds. When a view cell is activated by familiar view template, the view cell injects currents to the HD cells and the conjunctive grid cells, and activates a large number of cells to correct errors in velocity integration. On loop closure, the cognitive map is refined to further reduce the error caused by inaccurate motion.

1.2 Supplementary Video 2

A detailed example of network state calibration during loop closure by the help of visual feedback from the local view cells (ref. Figure 7). The calibration is achieved by the pattern completion mechanism of the attractor networks.
